# Supplementary material for: A multi-gene predictive model for the radiation sensitivity of nasopharyngeal carcinoma based on machine learning
Source: eLife. 2025 Jun 18;13:RP99849. doi: 10.7554/eLife.99849 (PMC12176387; doi:10.7554/eLife.99849)
Supplement: Supplementary file 1. [file elife-99849-supp1.docx]

| **Variable** |  | **Overall**(34) | **Res**(N=23 68%) | Radiosensitivity  **Sens**(N=11 32%) | **P value** |
| --- | --- | --- | --- | --- | --- |
| **Age**  Mean(SD)  Median[Min,Max]  **Sex**  Female  Male  **Clinical stage**  I  II  III  IV  **Pathology**  Undifferentiated  Poorlydifferentiated  Middle  Mix  Well |  | 46.38(11.7)  49.5[26,64]  9(26.47%)  25(73.53%)  3(8.82%)  4(11.76%)  6(17.65%)  21(61.76%)  22(64.71%)  5(14.71%)  1(2.94%)  3(8.82%)  3(8.82%) | 45.26(11.48)  49[26,61]  6(26.09%)  17(73.91%)  2(8.70%)  3(13.04%)  3(13.04%)  15(65.22%)  14(60.87%)  5(21.74%)  1(4.35%)  2(8.70%)  1(4.35%) | 48.73 (10.98)  50[31,64]  3(27.27%)  8(72.73%)  1(9.09%)  1(9.09%)  3(27.27%)  6(54.55%)    8(72.73%)  0(0.00%)  0(0.00%)  1(9.09%)  2(18.18%) | 0.406  0.942  0.824  0.326 |
