# Supplementary material for: A multi-gene predictive model for the radiation sensitivity of nasopharyngeal carcinoma based on machine learning
Source: eLife. 2025 Jun 18;13:RP99849. doi: 10.7554/eLife.99849 (PMC12176387; doi:10.7554/eLife.99849)
Supplement: Supplementary file 4. [file elife-99849-supp4.docx]

The data processing and analysis in this study were primarily conducted using the following software and tools. Raw transcriptomic sequencing data were quality-controlled and cleaned using **FastQC** and **Trimmomatic**, followed by alignment using **HISAT2**. Gene expression levels were calculated using featureCounts, and data were standardized using the **FPKM** method. GEO data (GSE32389) were based on the Affymetrix GPL570 platform and underwent background correction, quantification normalization, and log2 conversion using the affy package in R. Differential expression analysis was performed using the **limma** R package. For machine learning model construction, 12 algorithms, including LASSO, Ridge, Elastic Net, Stepglm, SVM, glmBoost, LDA, plsRglm, Random Forest, GBM, XGBoost, and Naive Bayes, were integrated and combined to form 113 model schemes, which were cross-validated and evaluated using the R programming language. Immune infiltration analysis was based on the **CIBERSORT** algorithm, and the relationship between key genes and immune cells was evaluated using Spearman correlation analysis. Single-cell RNA sequencing data were aligned and counted using **Cell Ranger** (v2.0.1) and **STAR**, and standardized, dimension-reduced, clustered, and differentially analyzed in the **Seurat** R package. Harmony was used for batch effect correction. Immune feature annotation was referenced from the **TISIDB** database, and cell subset annotation was performed using the **CellMarker** database and the **SingleR** R package. Pathway enrichment analysis included **GSVA** and **GSEA**, using the **MSigDB** gene set. Statistical analysis was performed in R language (v4.1.2), and graphical visualization was also performed using R.
